# Digital Biobanking and Big Data as a New Research Tool: A Position Paper

**DOI:** 10.3390/healthcare11131825

**Published:** 2023-06-22

**Authors:** Pamela Tozzo, Arianna Delicati, Beatrice Marcante, Luciana Caenazzo

**Affiliations:** Legal Medicine Unit, Department of Cardiac, Thoracic, Vascular Sciences and Public Health, University of Padova, 35121 Padova, Italy; arianna.delicati@phd.unipd.it (A.D.); luciana.caenazzo@unipd.it (L.C.)

**Keywords:** biobanking, big data, public health, healthcare governance

## Abstract

Big data analytics in medicine is driving significant change, as it offers vital information for improving functions, developing cutting-edge solutions and overcoming inefficiencies. With the right archiving and analysis tools, all players in the healthcare system, from hospitals to patients and from medical personnel to the pharmaceutical industry, can yield numerous benefits. Therefore, to analyze and interpret these analytics effectively, so that they can be useful for the advancement of scientific knowledge, we require information sharing, specific skills, training, integration between all system players, unique infrastructures and security. All these characteristics will make it possible to establish and harmonize real big data biobanks, for which it will be appropriate to consider new forms of governance compared to those traditionally conceived for large-sample biobanks.

## 1. Introduction

In the last few years, new digital technologies have led to a real turning point in many areas of daily life, especially in the areas of physical well-being, industry, safety and biomedicine [1,2]. Although they have been responsible for substantial benefits, the historical impact of these technologies has not been uniformly positive. Nevertheless, as a society, we place significant investment in prospective digital technologies to increase future wellbeing, while at the same time providing remedies for their accumulated negative impacts [3]. These new technologies have the potential to effect fundamental changes in our lives, from an environmental, social and commercial point of view, not only for their direct users but also for society in general [4,5].

The potential benefit of digital technologies used in medical research and practice should always be balanced against their potential risks, unexpected detrimental consequences and ambiguities [6]. These forms of harm may also occur on a public scale, either directly or indirectly, which individuals may not be aware of in order to opt out or choose to be excluded.

Newly established forms of biobanks are now involved in these new technologies, particularly when samples stored in these facilities are connected with electronic records from different areas that are assembled and may constitute the aggregation of big data, creating an opportunity to move beyond the use of population-level data for simple descriptive epidemiology to its use for making causal inferences.

An unformed mass of data in itself does not have great value for businesses, companies and research, but this can be assumed when such data are channeled through predictive algorithms, processing and interconnections, enabling one to analyze and predict the interests and consumption of individuals through profiling techniques and thus generate new data that are different from those initially provided by the data subject.

Data originating from citizens’ health information could gradually be accumulated from wellness and health-related apps, patients’ blogs, social networks, online shopping sites, etc. All these data may be of particular interest in epidemiology and public health, and this huge amount of information, if channeled with specific algorithms, could be used to identify target populations at greater risk of developing diseases or who could benefit more from some forms of primary prevention [7].

Many parameters relevant to human health, such as heart rate, blood pressure, glycemic control, level of physical activity, daily calorie intake, etc., can easily be collected from a variety of apps, which may even be free and readily available, or electronic devices. Technological advances, especially those related to the ability to process, accumulate and share data, have resulted in the proliferation of devices that make it possible for individuals to produce ever-larger streams of data across the lifespan, throughout the course of an illness, and in a specific geospatial context [8]. Starting with this large amount of data, it may be possible to develop new research models in the context of longitudinal studies using nominal current sources of the national health systems, transversal studies carried out by commercial and non-commercial packages that use nominal current sources of national health systems, analyses based on the use of non-nominal “big data”, healthcare profiles generated by initiatives carried out in the context of citizen science and personalized medicine based on personal devices.

In light of these premises and starting with the information that has been reported in literature thus far, the goal of our study is to provide a brief commentary that may be useful in the present field of research. In particular, in this study, we wish to highlight a conceptual contextualization of the establishment and the management of big data biobanks starting from the roots and to understand what directions these biobanks may take in the future.

## 2. Big Data Biobanking

Large amounts of data may be originated and collected in different ways [9,10]. Technology and big data procurement, if harnessed for the common good, could be the solution to some of the most pressing global challenges, including climate change, global pandemics and human trafficking. These data collections can represent autonomous forms of biobanks, traditionally conceived as collections of both samples and data, for which we will have to find dedicated forms of management, maintenance and regulation. These biobanks of data can be derived through different forms of procurement. However, since each country has a different healthcare system and different access rights, the utilization options may be very limited. Therefore, most of today’s medical data lack interoperability: the data are hidden in isolated silos and incompatible systems that are difficult to exchange, analyze and interpret [2,11,12]. Additionally, this slows down medical progress, as technologies that rely on these data cannot be used to their full potential [13]. Continuous interdisciplinary reflection is appropriate and indeed necessary, which sees different skills “talk to each other”, with the additional purpose of outlining the future structure of the multiple medical liabilities possibly connected with the use of new forms of biobanks and big data networks. Discovering the full potential of digital medicine requires an interconnected data infrastructure with fast, reliable and secure interfaces; international standards for data exchange; and medical terminologies that define unique vocabularies for information communication [14]. Interoperability, in the context of new networks of biobanks, may be useful for advances in digital health and should be a prerequisite for most of the innovations envisioned for future medicine [15].

### 2.1. Specific Health Information

This information is actively collected mainly in the context of specific epidemiological studies—within specific research hypothesis—or in the construction of biobanks of pathological records or biological samples, in which the referring population are selected from a single research project. In this context, it is important to provide clear indications on the ownership and use of data generated in research projects in relation to both intellectual property laws and privacy codes, paying particular attention to projects involving genetic data so that they are protected without affecting the efficacy of the research.

### 2.2. Health Information Spontaneously Generated by Health Systems

All this information is generated and archived electronically by the national health services whenever a service is provided to an assisted person, and therefore, the reference population represents all citizens who turn to public health services. The data contained in these archives are generally of two types: those referring to a pathological condition (identified by a code), such as hospital discharge cards, death certificates, ticket exemptions and electronic records related to the pathology; and those not directly identifying a pathology but identifying information such as drugs prescriptions. The information generated within public health systems is nominative in order to enable its use for diagnostic, therapeutic and evaluation purposes.

### 2.3. Health Information from Social Networks and Web-Based Systems

A particular case of the collection of health information through electronic and web-based systems is the so-called “Citizenveillance”, consisting of the sharing of personal data on a voluntary basis. This phenomenon may have specific hypotheses, as well as monitoring the health status of the community as it is structured. This type of system is favored by the spread of low-cost technology that allows for the measurement of health-related parameters, and the referring population is represented by all users of web-based services. It would certainly be appropriate to identify good practices for internet providers and to develop a code of conduct based on them, taking into account the relevant jurisprudence. When using big data integrated with health data, a specific risk assessment should be developed that takes into account the composition and characteristics of the group of users from whom the collected data are collected and the relationship between information about internet traffic and sensitive data (community privacy).

### 2.4. Socio-Economic Data

This type of data are collected and stored for socio-economic purposes that are linked to the subject and generated by several public institutions in the fulfillment of their aims. The referring population represents all members of the group considered (taxpayers, current account holders, citizens). It is evident that the possibility of using data from databases for different purposes may increase the potential and informativeness of data related to biological samples stored in biobanks when using them for research [16].

## 3. Big Data Management, Privacy Protection and Stigmatization Prevention

The rapid development of technologies has introduced new transformations reconfiguring traditional boundaries in the management of data: new configurations of technologies, service providers and users are challenging the existing regulatory categories, presenting novel opportunities and risks and raising important ethical questions [17,18]. The possibility of sharing large amounts of data on a large scale can create problems in terms of protecting the privacy and security of the people to whom those data belong and can create new interpretative solutions to more traditional issues, such as consent and respect for the principle of justice. Many big data sets belonging to research projects are collected and managed starting with a broad form of consent to the future multiple usages of these data for different research projects. Changing the paradigm from specific consent to broad consent to the use of research data may undermine the protection of research participants and may raise new questions about privacy protection [19]. Similarly, global, networked flows of data are also redefining the meaning of other traditional protections of human subject research, such as the right to withdraw from participation. Many new questions emerge from the possibilities provided by new technologies and the availability of large amounts of data. What are the implications of these changes for public trust and accountability in research? What governance options are afforded, and which capabilities are required for the digital and algorithmic processing of data on a global scale? Is there a danger that the increasing focus on individual biological and lifestyle causes of disease might overshadow efforts to address environmental and systemic determinants of illness?

As translational endeavors foster new kinds of engagement between physicians, researchers, patients, citizens, authorities and companies, it is important to consider how this affects what it means to be engaged in research [20,21]. Although individuals expressed concerns about maintaining their privacy, they conveyed considerable willingness to have their data shared with and used by researchers. It has been reported in the literature that even if citizens tend to be in favor of sharing their data, including health data, satisfying this need can be particularly complex due to the regulatory and legal constraints that this entails [22]. Often, there are issues related to the language used in the disclosures and terms of use for participants or technical issues related to the transmission and sharing of data between companies or between companies and researchers [23]. There is clearly a widespread need to standardize and simplify data sharing while still ensuring the security of the people who share these data.

Confidentiality safeguards with respect to data collection in medical records, ongoing human clinical trials and public health surveillance policies may not be adequate given the ever-increasing amount of data being produced and collected [24].

Often, the users of health-related applications are not fully aware of the terms of use of the data they provide and of the strategies for protecting their privacy, with unpredictable effects on possible damages to the protection of citizens’ privacy [25]. The greatest risk that can result from the accumulation of these large quantities of data is related to the possibility that, by combining several sources, one can arrive at the identification of the subject to whom those data belong [26]. It would be useful to act on several levels (regulatory, legal, technological) in order to standardize procedures that allow for the protection of confidentiality when using these large amounts of data for research, with the aim of protecting the common good and avoiding violations and inappropriate uses. At the same time, it would be useful to better understand citizens’ preferences and opinions on these issues, their willingness to provide data and their concerns about the inappropriate use of their data. Greater involvement of the public is also desirable, both to help people to understand the possibilities provided by the use of big data and to encourage discussion on problematic issues, such as privacy control and security. Moreover, the ability to provide citizens with tools to monitor research and to exercise some form of control, too, could be a useful tool in implementing these forms of biobanks. These transformations are reconfiguring traditional boundaries between the public domain (healthcare systems, medical research and clinical practice) and the private one (patients and citizens) [27].

## 4. Public Trust and Big Data Management

Although the last decade has seen the emergence of principles reflecting shared ethical assumptions that foster international scientific collaborations, the idea that sharing and exchanging data in biobanks serve as a form of altruism and solidarity is still evolving [28]. Progress in research can also be achieved through the greater awareness of citizens and by ensuring their trust in researchers, both through transparent language and by improving the protection and control systems used to protect citizens from the indiscriminate and counterproductive use of their data [29]. When we discuss public involvement in science and technology, we mean the set of strategies, initiatives and tools that are used to activate reciprocal relationships between institutions, researchers, organizations and the public with the aim of generating common benefits. Furthermore, we should develop approaches to foster trust related to specific contexts with which research will interface, including, big data collection, management and sharing [30]. Public communication and public consultation should be enhanced in order to educate stakeholders and citizens and inform decision makers about public opinion on specific topics of interest in research and innovation in big data biobanks. Participatory activities with the involvement of all the stakeholders, particularly the general public, should be performed with the aim to reach, for example, web-based discussion platforms. This type of multimedia toolkit with interactive resources may be helpful for running awareness campaign through social media and dedicated digital webinars.

As with earlier debates in bioethics, much attention in clinical practice has centered on the patient, focusing on informed consent, privacy protections and the risks of exploitation. Important as these concerns are, the time has come to update the ethical/legal dialog in regard to the collection of large amounts of data for research purposes to accommodate broader social and political perspectives.

Security in the use, sharing and transfer of data should be achieved through the implementation of shared procedures and operational guidelines that are applicable for researchers regardless of their geographical location. In addition, it is important to build the mutual trust that is required for the delegation of ethics reviews across jurisdictions, where such delegation is feasible and appropriate.

The editorial policies of scientific journals, which impose conditions such as transparency on these issues and compliance with the requirements used to protect the confidentiality of data relating to participants in biomedical research, are important steps in this direction [31].

In order to implement networking in the collection of high-quality data for use in a wide range of research projects, it is also important to create an institutional environment sensitive to these issues. Indeed, strengthening relationships between governments, industry and the general public is of paramount importance for creating and maintaining such an environment. With this objective, it would be useful to invest in structured policies for effective education and information for citizens. Despite the high and necessary costs for the creation and maintenance of these structures, the conscious participation of citizens could certainly favor the progress of both individual and public health [32,33,34,35].

It is a duty of both public and private research institutions to take into account the opinions and expectations of the public, as well as institutional needs, in terms of health policies, through transparent and up-to-date information on their research activities, especially when large amounts of data are used. An important element of the infrastructural development of these new forms of biobanks that collect big data involves substantial investments used to ensure access to data that are secure, ethically oriented and useful. It is also desirable to promote public debate on the developments and limits of the use of big data in medicine, so that it will be possible for all citizens to acquire the elements of “big data literacy” in order to actively participate in social discussions. These are the essential premises for the possibility of overcoming the “digital divide” in medicine, promoting greater inclusiveness.

The main objectives that we should set ourselves are as follows:

- To understand the state-of-the-art research in relation to the dissemination, structuring, implementation and management of public engagement programs in big data management and research in order to gain insight concerning which implementation patterns can truly facilitate inclusive and participated research and innovation where big data are concerned;
- To implement inclusive actions fostering public engagement that consider a multitude of views, enhancing the manifestations of a socially oriented and sustainable research and innovation future that is compatible with international, European and national policies, thus fostering public communication and public consultation;
- To bring society a step closer to biobank infrastructures, linking, where possible, research and innovation to largescale societal challenges through public deliberation and public participation, establishing confidence in the use of big data within research;
- To develop new tools and digital public engagement infrastructures for everyone interested in using citizen science and enable the discussion and co-production of ideas.

## 5. Conclusions

Creating systems that seamlessly link research to clinical care is perhaps the greatest challenge in the field of biomedical sciences today. For this long-term goal, it is necessary to consider a more sophisticated data ecosystem and find ways to use the large amounts of existing data in new, innovative models, incorporating data from heterogeneous sources to create new value in health services and in scientific research, thus providing new forms of biobanking infrastructures.

Specific and dedicated solutions should balance respect for the rights of the individuals to whom the data belong with the need to implement responsible research projects. It is therefore advisable that developers, managers and even users of big data carefully monitor the evolution of legislation in order to guarantee the compliance of the related infrastructures and to enable adequate risk management. There is also work to be done to ensure that policy, legal and technological developments enhance the potential to generate knowledge from biobank-related big data, and it will be of paramount importance to train processes for third-millennium health personnel and modern, cultured managers with a vision of public health that is not merely managerial.

## Data Availability

For this manuscript, no new data were created.
